# The Combination of* Ephedrae herba* and* Coicis semen* in Gambihwan Attenuates Obesity and Metabolic Syndrome in High-Fat Diet–Induced Obese Mice

**DOI:** 10.1155/2018/5614091

**Published:** 2018-08-19

**Authors:** Jun-Woo Jang, Dong-Woo Lim, Ji-Ung Chang, Jai-Eun Kim

**Affiliations:** Department of Pathology, College of Korean Medicine, Dongguk University, Goyang 10326, Republic of Korea

## Abstract

Gambihwan is a herbal prescription used in Korean medicine to treat obesity. The authors evaluated the effects and mechanisms of two types of Gambihwan (GBH1 and 2) administered to high-fat diet– (HFD-) induced obese mice. Four-week-old C57BL/6 mice were fed a HFD for 8 weeks with or without GBH1 or 2 (100-200 mg/kg/day by oral gavage). All mice were subjected to glucose tolerance testing after the 8-week treatment period and then euthanized. Serum insulin, lipids, and inflammatory cytokine levels were analyzed using commercial kits. Hepatic enzyme levels and lipid profiles were also investigated. Liver section slides were stained with Oil Red O (ORO) or hematoxylin and eosin (H&E) to assess lipid accumulation. GBH1 and 2 both significantly decreased body, liver, or adipose tissue weights in HFD-fed mice and significantly improved glucose tolerance (p<0.05 in all groups). Cholesterol levels in both sera and liver homogenates were significantly decreased by GBH1 and 2 (p<0.05 in all groups). In addition, serum inflammatory cytokines (p<0.05 in 200 mg/kg/day groups) and hepatic enzyme levels were significantly diminished by GBH administration at 200mg/kg/day (p<0.05 in all groups). Furthermore, histologic analyses of liver sections revealed GBH suppressed lipid accumulation. Both GBH types suppressed HFD-induced increases in body weight and obesity-related markers in HFD-fed mice despite the difference in constituents between GBH1 and 2. It is strongly assumed that the combination of* Ephedrae herba* and* Coicis semen* exerted the antiobesity effect. The results obtained show that the antiobesity effects of GBH warrant further investigation.

## 1. Introduction

Obesity is characterized by the accumulation of excessive body fat [1] and in Asia is defined as a body mass index (BMI) of > 25 kg/m^2^ [2]. Because of its dramatically increased prevalence worldwide [3], the prevention of obesity is viewed as an issue of considerable importance [4]. Various factors contribute to the development of obesity in man, such as, food, environment, lifestyle, drugs, and endocrinal disorders [5]. Among these factors, changes in dietary habits, particularly with respect to the increased intakes of fat, caloric sweeteners, and animal sourced food, are considered to be major causative factors of the recent obesity pandemic [6].

Sibutramine was withdrawn from the market in 2010 because it was found to increase cardiovascular event rates [7], and, as a result, the antiobesity drugs prescribed for chronic administration (>1 year) in Korea are Lorcaserin and Orlistat [8]. Lorcaserin is an authorized serotonin receptor agonist that reduces body weight by reducing food intake [9], while Orlistat is a lipase inhibitor that reduces dietary fat absorption [10]. However, Orlistat has been reported to have gastrointestinal side effects [11], and users have complained that Lorcaserin causes nausea and vomiting. Because of these reported side effects, numerous herb-derived agents believed to reduce obesity are being investigated with a view toward developing safer and more effective agents [12].

Various herbal prescriptions are used in clinics to treat a broad spectrum of obese or overweight patients [13]. Gambihwan (GBH) is a Korean herbal medicine that is prescribed to treat obesity. Prescriptions are composed of major constituent herbs of Bangpungtongseong-san (Bofutsusyosan) and Taeeumjowitang, which are widely used in Korean medicine to treat obesity [14, 15]. Modification in composition of GBH makes its indication more clear with Sasang constitutional medicine. GBH1 is more suitable for So-Yang pattern obesity in Sasang constitutional medicine and GBH2 is more appropriate in treating Tae-eum pattern obesity. So-yang type patients with thick subcutaneous fat, strong abdomen elasticity, and constipation are recommended to take GBH1 [15]. On the other hand, Tae-eum type patients with headache and stomachache, diarrhea, and chest congestion (caused by food intake) are recommended to take GBH2 [16]. Although the safety and efficacy of GBH in obese patients have been demonstrated by clinicians [17], its mechanisms and effects have not been determined* in vitro *or* in vivo*.

High-fat diet– (HFD-) induced obese mice are a widely used animal model and display the characteristics of weight gain, hyperlipidemia, hyperglycemia, hyperleptinemia, insulin resistance, and systematic low-grade inflammation associated with obesity in man [18, 19]. Because it is composed of several medicinal herbs which possess beneficial effects against obesity, GBH might manage these symptoms of obesity by exerting multidirectional effects.

In this study, we investigated suppression of the symptoms of obesity in HFD-induced obese mice fed two different GBH prescriptions (GBH1 and 2), and compared the efficacies of these two prescriptions. Therefore, we demonstrated the antiobesity effect of GBH administration in vivo and tried to build grounds for the usage of GBH in clinics.

## 2. Materials and Methods

### 2.1. Preparation of Herbal Prescriptions

The herbal prescriptions GBH1 and GBH2 used in this study were prepared by hot-water extraction. The herbal formulae of GBH1 and GBH2 are provided in Table 1. Briefly, dried raw materials were mixed and boiled in 2 L of distilled water for 1.5 h, and the crude extract was then obtained by filtration through Whatman No. 4 filter paper, transferred to a rotary evaporator (Buchi, Flawil, Switzerland), and concentrated at 95°C. The extract so obtained was then frozen and freeze-dried over 3 days to obtain powdered extracts (GBH), which were stored at -20°C and dissolved at distilled water for administration.

### 2.2. Animals

Forty-eight, 4-week-old male C57BL/6 mice purchased from Orientbio (Gyeonggi-do, Republic of Korea) were kept in semi-pathogen-free (SPF) animal care unit maintained at 20 to 22°C and a relative humidity of 55%. Mice were housed in 12 cages, four mice per cage, and acclimatized after receipt for 7 days. They were then randomly allocated to one of 6 groups, as follows: a normal diet (ND) group, a high-fat diet (HFD) group, a high-fat diet + low dose GBH1 (GBH1 LD) group, a high-fat diet + high dose GBH1 group (GBH1 HD), a high-fat diet + low dose GBH2 group (GBH2 LD), or a high-fat diet + high dose GBH2 group (GBH2 HD). The HFD (60% kcal fat) was purchased from Saeronbio (Gyeonggi-do, Republic of Korea). GBH groups were administered GBH at low (100 mg/kg/day) or high doses (200 mg/kg/day) for 8 weeks (5 days per week) by oral gavage. Body weights and food intakes were measured weekly. After the 8-week treatment period, mice were starved for 12 h and euthanized for analysis. Blood was collected and centrifuged at 3000 rpm for 20 min to obtain serum. Organs were weighed and collected in separate tubes. All samples were stored at -80°C before analysis. The protocols of animal experiments were approved beforehand by the Ethics Committee of Dongguk University (No: IACUC-2017-005).

### 2.3. Oral Glucose Tolerance Test (OGTT)

At the end of the 8-week treatment period, mice were subjected to OGTT. Mice were starved for 12 h before measuring fasting glucose levels. All mice were administrated 1.5 g/kg of glucose and blood glucose levels were determined 0, 30, 60, 90, and 120 min later using a blood drop taken from the ends of tails. Blood glucose levels were measured using an instant glucose meter and disposable strips (ACCU-CHEK Active, Roche Diagnostics, USA).

### 2.4. Serum Lipid Analysis

Lipids in sera were analyzed to determine the efficacy of GBH. Serum triglycerides and total cholesterol levels were determined using a commercial colorimetric assay kit (Asan Pharmaceutical, Seoul, Republic of Korea) using whole serum samples as the provided standard. Mouse serum samples were centrifuged to precipitate low density lipoprotein for separation, and high density lipoprotein (HDL) levels were measured in supernatants. Total cholesterol levels were determined by measuring optical densities at 500 nm, and triglyceride levels were measured at 550 nm.

### 2.5. Liver Lipids Analysis

Total lipids in liver tissues were extracted using Folch's method [20] with slight modification. In brief, the same weights of mouse livers were sliced and homogenized. For each sample, a chloroform and methanol (2:1, v/v) mix was added to liver homogenate and vortexed, and the homogenate was then capped and incubated at RT for 48 h to extract lipids. A portion of the bottom fraction was then extracted using syringe, transferred to a new tube, and dried completely in a fume hood. Finally, extracted total lipids were resuspended in 2-propanol and analyzed using commercial kit (Asan Pharmaceutical, Republic of Korea).

### 2.6. Serum Hepatic Enzyme Level Analysis

Liver enzyme (glutamate oxaloacetate transaminase and glutamate pyruvate transaminase) levels in mouse sera were used as markers of liver damage caused by excessive lipid accumulation. Mouse serum samples were analyzed using a colorimetric assay kit (Asan Pharmaceutical, Republic of Korea). Absorbances were read in 96-well plates at 505 nm and kit standards were used as calibration controls.

### 2.7. Serum Inflammatory Cytokine Analysis

Inflammatory cytokines were analyzed using a commercial enzyme-linked immunoassay (ELISA) kit (Thermo Fisher Scientific, Waltham, MA, USA) including streptavidin-HRP and TMB substrate solution. Tumor necrosis factor *α* (TNF*α*), interleukin-1 beta (IL-1*β*), and interleukin-6 (IL-6) levels in mice sera were measured. The absorbances were measured using microplate reader at 450 nm within 30 min of final reaction.

### 2.8. Histology

For histologic analysis, mouse liver tissues were sliced, embedded onto a specimen disc in frozen section media (FSC 22 compound, Leica, Jena, Germany), and frozen at -20°C. Liver tissue section slides were prepared at a thickness of 8 *μ*m using CM1860 cryostat microtome system (Leica Biosystems, Nussloch, Germany). Sliced liver sections were placed on slide glasses, air-dried for 1 day, and stained with hematoxylin (Mayer's hematoxylin) and eosin (H&E). In addition, sections were separately stained with Oil Red O and hematoxylin to assess lipid deposition.

### 2.9. Statistical Analysis

Results are expressed as means ± standard deviations (SD) and analyzed using SigmaStat 3.5 (Systat Software, CA, USA). The Student's t-test and one-way ANOVA with Bonferroni* post hoc* test were used to determine the significance of differences between groups. P values of < 0.05 were considered statistically significant. Microsoft Excel was used for the regression analysis. Figures and tables were produced using Graph Pad Prism 5 (Graph Pad, La Jolla, CA, USA).

## 3. Results

### 3.1. GBH Administration Significantly Decreased Body and Organ Weights

**Figure 1:**
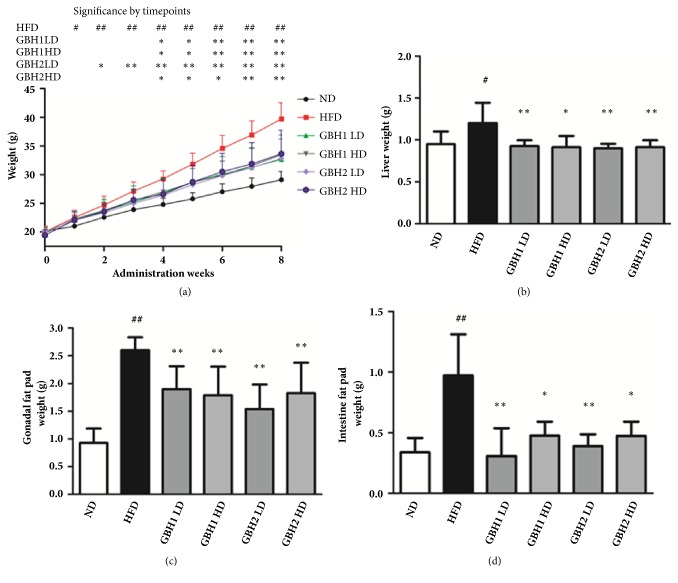
Body weight and organ weight of all groups after GBH administration for 8 weeks. (a) Weekly body weight changes after administration of herbal prescription. (b) Liver weight. (c) Gonadal fat weight. (d) Intestinal fat weight after 8-week administration. # p < 0.05 and ## p <0.01 versus ND group; *∗* p < 0.05 and *∗∗* p < 0.01 versus HFD group.

Body weights were measured weekly to determine the effect of GBH on obesity. Body weights in the HFD group increased significantly more than in the ND group (Figure 1(a)). However, the oral administrations of GBH1 or GBH2 (high or low dose) significantly inhibited body weight increases (p<0.05) as compared with the HFD group from the 4^th^ week to the end of the 8-week treatment period. Liver and fat weights were significantly decreased by GBH1 or GBH2 administration but not in dose-dependent manner (Figures 1(b), 1(c), 1(d)).

### 3.2. GBH Administration Attenuated Glucose Homeostasis and Reduced Serum Insulin Levels

**Figure 2:**
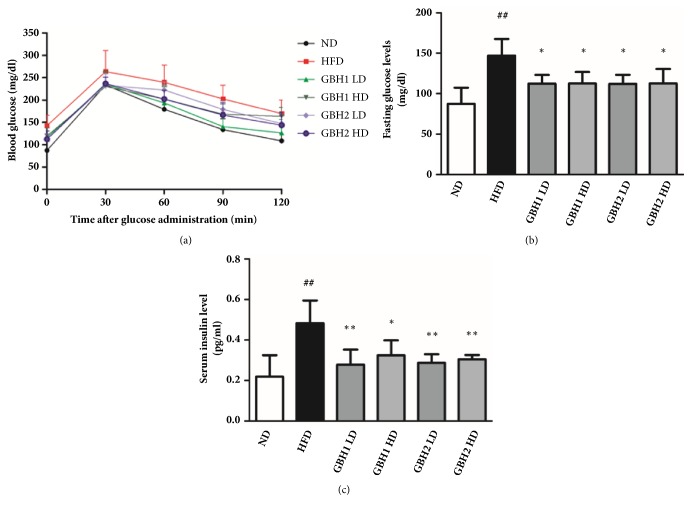
Parameters related to glucose metabolism: (a) oral glucose tolerance test, (b) fasting glucose level, (c) serum insulin level. # p < 0.05 and ## p <0.01 versus ND group; *∗* p < 0.05 and *∗∗* p < 0.01 versus HFD group.

Oral glucose tolerance testing conducted after the 8-week treatment period showed that the HFD group had significantly higher glucose levels (p<0.05) than the ND group at 0, 60, 90, and 120 min point after glucose challenge (Figure 2(a)). However, groups administrated GBH1 or GBH2 had lower average blood glucose levels than the HFD group at all time points (0, 30, 60, 90, and 120 min). In addition, blood glucose levels were lower in the four GBH1 or GBH2 groups than in the HFD group at 60 and 90 min after glucose administration. Fasting glucose levels were also increased by HFD feeding, but the administrations of GBH1 or 2 significantly reduced (p<0.05) these glucose levels (Figure 2(b)). Similarly, serum insulin levels were markedly higher in the HFD group than in the ND group, but lower in the GBH1 or 2 groups than in the HFD group (Figure 2(c)).

### 3.3. GBH Administration Modulated Serum Lipid Profiles

**Figure 3:**
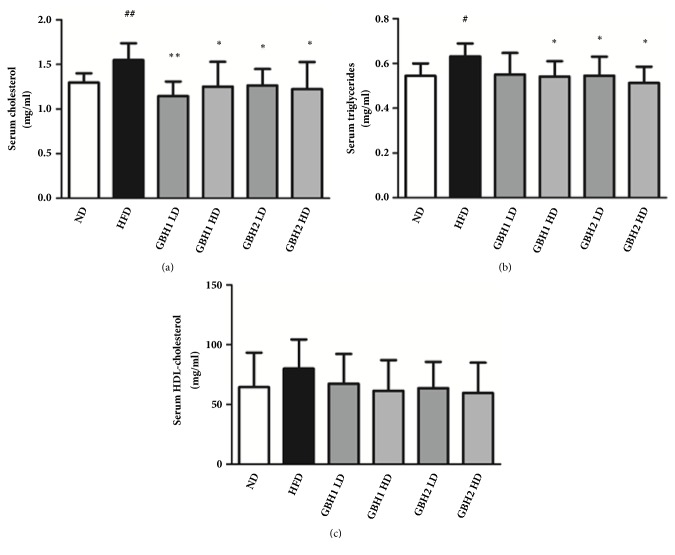
Lipid profiles of mice serum analyzed after 8 weeks of administration: (a) total cholesterol, (b) triglycerides, (c) high density lipoprotein. # p < 0.05 and ## p <0.01 versus ND group; *∗* p < 0.05 and *∗∗* p < 0.01 versus HFD group.

Total serum cholesterol was increased by HFD feeding, as previously reported [21] (Figure 3(a)). Serum triglycerides were significantly higher in the HFD group (p<0.05) than in the ND group (Figure 3(b)). However, serum triglyceride and total cholesterol levels (except serum triglycerides in the GBH1 high dose group) were markedly lower in the four GBH groups than in the HFD group. On the other hand, neither HFD nor GBH administration caused significant changes in serum HDL cholesterol levels (Figure 3(c)).

### 3.4. GBH Administration Decreased Serum Inflammatory Cytokine Levels

**Figure 4:**
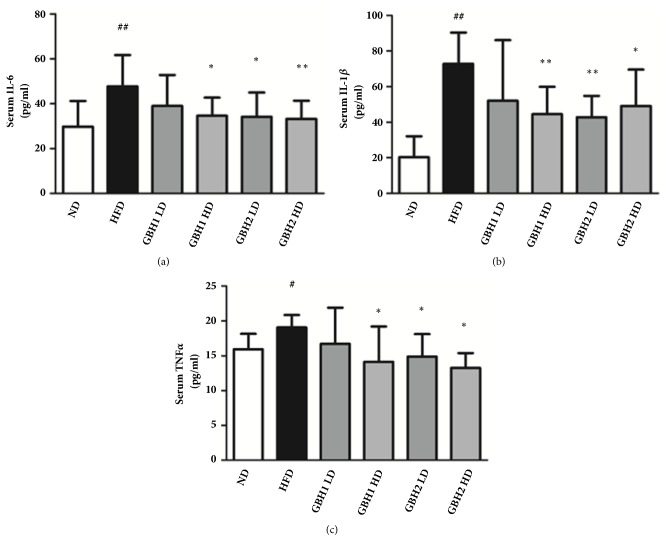
Inflammatory cytokines of mice serum determined by ELISA kit: (a) IL-6, (b) IL-1B, (c) TNF*α*. # p < 0.05 and ## p <0.01 versus ND group; *∗* p < 0.05 and *∗∗* p < 0.01 versus HFD group.

Levels of the inflammatory cytokines IL-6, IL-1*β*, and TNF*α* were measured to determine the effect of GBH on systematic inflammation induced by HFD. Serum IL-6, IL-1*β*, and TNF*α* levels were significantly increased by HFD (by 1.60-, 3.59-, 1.20-fold, respectively; p<0.05) (Figures 4(a), 4(b), 4(c)), and these increases were markedly reduced by high dose GBH1 and high and low dose GBH2 administration (p<0.05). This effect was found to be dose-dependent (Figure 4(b), p<0.05), with the exception of the GBH1 LD group, in which HFD-induced increases in inflammatory cytokine levels were not suppressed.

### 3.5. GBH Administration Reduced Hepatic Enzyme Levels in Serum

**Figure 5:**
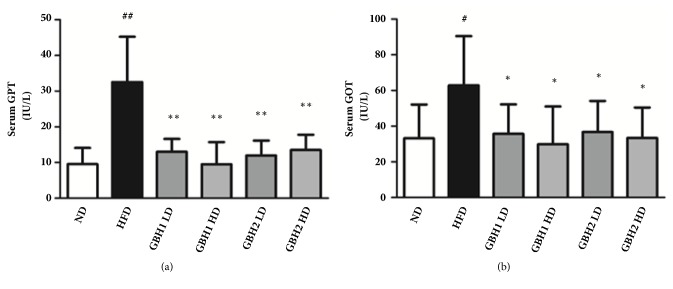
Hepatic enzyme levels of mice serum after 8 weeks of administration. (a) Glutamate pyruvate transaminase (GPT). (b) Glutamate-oxalacetate transaminase (GOT). # p < 0.05 and ## p <0.01 versus ND group; *∗* p < 0.05 and *∗∗* p < 0.01 versus HFD group.

Hepatic enzyme activities in serum were measured to assess protective effect of GBH on liver damage caused by excessive lipid accumulation. After the 8-week treatment period, serum GPT and GOT levels were significantly higher (3.42- and 1.89-fold, respectively) in the HFD group than in the ND group (Figures 5(a) and 5(b), p<0.05). However, all four GHB administered groups showed significant reductions in GPT and GOT levels as compared with the HFD group (p<0.05). Moreover, serum GOT levels were found to be dose-dependently reduced by GBH1 and GBH2.

### 3.6. GBH Administration Decreased Liver Lipid Contents

**Figure 6:**
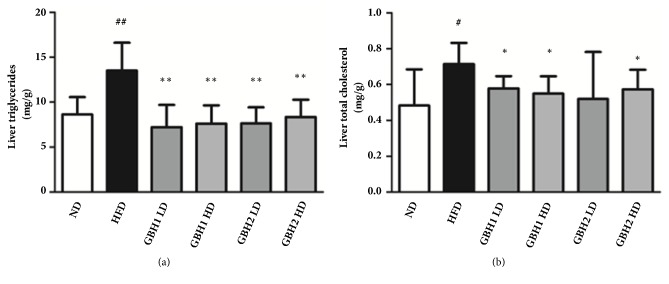
Lipid profiles of mice liver after 8 weeks of administration: (a) triglycerides, (b) total cholesterol. # p < 0.05 and ## p <0.01 versus ND group; *∗* p < 0.05 and *∗∗* p < 0.01 versus HFD group.

Extracted triglyceride and total cholesterol levels in liver were both noticeably higher in HFD group than in the ND group (Figures 6(a) and 6(b), p<0.05). However, the four GBH treated groups had significantly lower lipid levels (except total cholesterol level in the GBH2 LD group, Figures 6(a) and 6(b), p<0.05), though no dose-dependent manner was observed.

### 3.7. GBH Administration Reduced Fat Deposition of Liver

**Figure 7:**
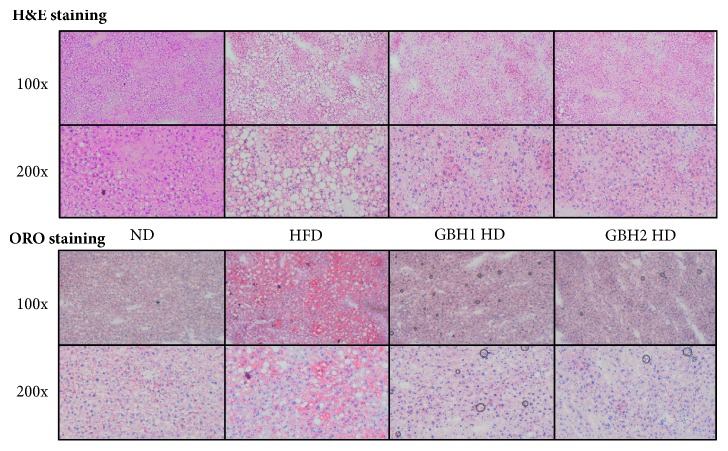
Histologic analysis of mice liver and adipose tissue after 8 weeks of administration, (upper) hematoxylin & eosin staining of liver section slides, (below) Oil Red O staining of liver section slides.

Liver sections were stained with Oil Red O or H&E to examine the effect of GBH administration. Histologic analysis of H&E stained sections revealed excessive lipid accumulation in livers of HFD-fed mice and notable increase in the areas of nonstained tissues due to lipid accumulation (Figure 7 upper). However, stained sections from the GBH1 and GBH2 groups showed less lipid accumulation. Similarly, Oil Red O staining of liver sections showed definite increases in lipid droplets in the HFD group (Figure 7 below) and apparent decreases in these stained areas in the four GBH1 or GBH2 administered groups, suggesting the inhibition of lipid accumulation in liver.

## 4. Discussion

The present study describes the antiobesity effects of GBH in an obese mouse model. The administrations of GBH1 or GBH2 were found to have significant weight-loss effects.* Ephedra sinica*, also called Ma Huang in Korean medicine, was reported to cause notable weight loss in clinical study, and this was attributed to the presence of ephedrine (a phenethylamine alkaloid) [22]. In combination with the xanthine alkaloid caffeine, ephedrine can promote thermogenesis by activating the sympathetic nervous system which results in increased energy expenditure [23]. Thus, it seems that ephedrine might have exerted similar weight-loss effects in the present study.

Impaired glucose metabolism is another characteristic of obese subjects [24], and glucose clearance rate is used as an index for estimating glucose homeostasis [25]. In the present study, GBH administration was found to regulate glucose homeostasis by controlling blood glucose levels and insulin levels in mice. Along with the well-known beneficial effects of* Ephedra sinica* on glucose intolerance [26], the seeds of* Coix lacryma-jobi* (*Coicis semen*) have also been reported to have hypoglycemic activity in a db/db diabetic mouse model [27], and this was presumed to be due to the presence of coixans and glycans [28, 29].

Low-grade systematic chronic inflammation is frequently observed in obese subjects. Diet-derived saturated fatty acids can induce toll-like receptor– (TLR-) mediated inflammatory response and increase the levels of proinflammatory cytokines, including those of IL-6, IL-1*β*, and TNF-*α* levels [30]. It has been reported that some materials in GBH possess anti-inflammatory effects. Polyphenols from* Arecae semen* (a component of GBH1) have been suggested to exert anti-inflammatory effects against gastrointestinal inflammation [31], and baicalin, a major bioactive compound found in* Scutellariae radix*, has been reported to have anti-inflammatory properties [32], which could explain the marked decrease in inflammatory cytokines levels observed in the two GBH2 fed groups.

Excessive fat intake derived from the HFD feeding often leads to hepatic steatosis [33], which induces mild to severe grade liver injury and histological changes [34]. In the present study, HFD-fed mice showed increased hepatic enzyme levels and elevated hepatic lipid levels, which are consistent with other studies. However, GBH1 and GBH2 successfully protected livers from damage and significantly maintained parameters in the normal range. This was further supported by histological analysis of liver slides, as fewer and smaller lipid vesicles were observed in GBH groups than in the HFD group. Along with reduced lipid profiles in mice serum, it is presumed that GBH1 and 2 exert hypolipidemic effects by systemic mechanism. In addition, extracts from* Coicis semen* and* Ephedrae herba* are reported to have hypolipidemic effects on obesity models [35, 36] similar to presented results.

Several authors have suggested that dietary supplementation of* Ephedra sinica* extract can cause severe adverse effect on the cardiovascular system including hypertension, palpations, tachycardia, and even seizure or death [37]. Therefore, dosages of herbal prescriptions including* Ephedra sinica* should be carefully considered in a clinical setting. However, in the present study, no serious side effect was observed in any of the GBH fed groups, which indicates that our prescription is safe at a dosage of 200 mg/kg/day in our mouse model. This is consistent with the result of a previous study, in which no mortality was reported as a result of* Ephedra sinica* extract administration at dosages below 289 mg/kg daily [38].

*Ephedrae herba* and* Coicis semen* are common, major constituent herbs of GBH1 and 2. A review study reported that the combination of the two herbs is most frequently used herbal combination in recent studies on obesity [39]. The combination of two herbs can be frequently found in other antiobesity studies [40–43]. Concerning the theories of traditional formulation [44], the combination assumed to play major role in antiobesity properties of GBH1 and 2.

We conclude GBH should be considered as potential herbal antiobesity agent and recommend further studies be conducted to elucidate the mechanisms responsible for the antiobesity effect of GBH and to identify the active compounds in GBH. Moreover, extensive investigation should be performed on antiobesity effect of the herb combination in further studies.

## 5. Conclusions

GBH is a herbal prescription in Korean medicine consisting of* Ephedrae herba* and* Coicis semen* as major constituents to reduce obesity. In the present study, 8 weeks of GBH1 or 2 administration significantly decreased body weights and organ weights in our HFD-fed mouse model of obesity despite the difference in constituents between GBH1 and 2. Moreover, GBH administered mice showed improved glucose tolerance and lipid profiles. GBH administration was also found to have significant suppressive effects on serum inflammatory markers and attenuated liver steatosis. It is strongly assumed that the combination of* Ephedrae herba* and* Coicis semen* exerted the antiobesity effect. Overall, our findings indicate that GBH is worthy of further investigation as a potential antiobesity agent.

## Figures and Tables

**Table 1:** Herbal composition of modified GBH 1, 2.

GBH1		GBH2	
Herbs	Content (g)	Herbs	Content (g)

*Ephedrae Herba*	24	*Ephedrae herba*	36

*Coicis semen*	36	*Coicis semen*	54

*Menthae herba*	24	*Typhae pollen*	54

*Gypsum*	36	*Castaneae semen*	18

*Alismatis Rhizoma*	18	*Sinomeni Caulis et Rhizoma*	18

*Crataegi fructus*	18	*Scutellariae radix*	18

*Arecae semen*	18		

*Hordei fructus germinatus*	18		

Total weight	192		198

Yield	30.61		28.92

## Data Availability

The data used to support the findings of this study are included within the article.

## Abbreviations

BMI: Body mass index
GBH: Gambihwan
HFD: High-fat diet
SPF: Semi-pathogen-free
OGTT: Oral glucose tolerance test
HDL: High density lipoprotein
GOT: Glutamate oxaloacetate transaminase
GPT: Glutamate pyruvate transaminase
ORO: Oil red o
ELISA: Enzyme-linked immunoassay
TLR: Toll-like receptor.
